# Passive Movement Exercise of the Lower Limbs May Facilitate Wound Healing in Patients With Diabetic Foot Ulcers

**DOI:** 10.7759/cureus.73925

**Published:** 2024-11-18

**Authors:** Tue Smith Jørgensen, Amalie Schramm, Maria Leinum, Hans Gottlieb, Stig Brorson, Ylva Hellsten, Birgitte Høier

**Affiliations:** 1 Department of Orthopedic Surgery, Herlev Hospital, Herlev, DNK; 2 Department of Plastic and Reconstructive Surgery, Herlev Hospital, Herlev, DNK; 3 Department of Nutrition, Exercise and Sports, Faculty of Science, University of Copenhagen, Copenhagen, DNK; 4 Department of Orthopedic Surgery, Zealand University Hospital, Køge, DNK

**Keywords:** diabetic foot ulcer (dfu), passive movement, peripheral arterial disease (pad), wound care treatment, ­wound healing

## Abstract

Background: Diabetic foot ulcers are a frequent and serious complication of diabetes with a high risk of amputation. Exercise has been shown to promote wound healing; however, patients with non-healing foot ulcers have limited ability to exercise due to the foot ulcer. Other strategies are therefore warranted.

Methods: We evaluated the effect of eight weeks of two-leg passive movement exercise on wound healing in patients with non-healing diabetic foot ulcers. Twenty-one patients were included in the study and randomized into either a control or a passive movement exercise intervention group. The primary outcome measure was the wound area.

Results: Sixteen participants completed the trial. Wound sizes for the passive movement intervention group were 274 mm^2^ and 58 mm^2^ at baseline and week 8, compared to 148 mm^2^ (p=0.31) and 136 mm^2^ (p=0.51) in the control group (week 16; 7 mm^2^ vs. 23 mm^2^, p=0.55). The mean wound area percentual reduction between baseline and week 8 was higher in the intervention group (76% vs. 36%, difference 40%, p=0.062).

Conclusion: The two-leg passive movement intervention showed a non-significant difference in wound healing and was well tolerated by patients with diabetic foot ulcers. Although the study shows potential, the results should be interpreted with its limitations of being underpowered and potentially confounded. We encourage larger randomized controlled trials to be conducted, to elucidate whether the two-leg passive movement intervention can be used to accelerate wound healing in non-healing ulcers.

## Introduction

The incidence of diabetes has been rising exponentially during the last three decades [1]. Diabetic foot ulcers are one of the most frequent and serious complications of diabetes. Yet, knowledge regarding efficient treatment and the multifactorial causes of impaired healing in non-healing diabetic foot ulcers is limited [2-4]. Despite attempts at prevention and treatments, the amputation risk of diabetic patients is four times higher than the background population [5,6]. Moreover, foot ulcers are known to reduce the quality of life for patients physically and psychologically [7,8] and have major socioeconomic consequences for the patients and society [9,10]. Management of diabetic foot ulcers requires in-depth knowledge of the major risk factors for amputation, frequent evaluation, and thorough preventive maintenance. 

Up to 50% of patients with diabetes and foot ulcers have peripheral arterial disease (PAD) [11]. PAD is one of the main risk factors for the development of foot ulcers, in parallel with factors such as pressure load, neuropathy, and infection [12]. Exercise has been shown to promote wound healing in rodents with diabetes [13], and active exercise seems to increase wound healing in patients with diabetic foot ulcers [14,15]. But patients with non-healing foot ulcers are often limited in their exercise patterns due to immobilization and reduced functional physical abilities [16]. A recent review concluded that protective strategies are often preferred over therapeutic exercise that might have unforeseen consequences for patients over time [17]. Thus, an intervention that can increase blood flow and oxygenation to the limb, such as passive movement, could be an alternative first step for improving limb microcirculation that could lead to an enhanced potential for wound healing.

Passive movement of the lower leg has been shown to initiate angiogenesis from the skeletal muscle [18,19]. This effect is primarily due to enhanced mechanical signaling, as passive leg movement increases blood flow and passive stretch of the muscle with a negligible increase in muscle metabolism [20]. In young healthy individuals, four weeks of repeated passive movement have been shown to upregulate angiogenic factors and induce a limited enhancement in capillarization in the skeletal muscle [18]. In diabetic patients with foot ulcers, limited mobility in combination with poor blood flow and vascularization causes low angiogenic signaling via shear stress, stretch, and metabolism [21,22].

In this study, we hypothesize that an intervention with repeated passive movement will enhance blood flow and provide an angiogenic stimulus that will improve the microcirculation in these patients [19], thereby increasing the potential for wound healing.

We assessed the effect of a prototype for passive leg movement developed at the Department of Nutrition, Exercise and Sports, University of Copenhagen, in Copenhagen, Denmark [18,20].

The study aimed to evaluate the effect of eight weeks of passive movement sessions of both legs on wound healing in non-healing diabetic foot ulcers. We hypothesized that the intervention group would have a greater wound area reduction than the control group.

## Materials and methods

Overall study design

We conducted a pragmatic assessor-blinded randomized controlled clinical trial to compare wound healing in patients with non-healing diabetic foot ulcers. The patients were randomized into one of two groups: either standard wound treatment care alone or passive training and standard wound treatment care combined. The participants and the responsible recruitment doctor were not blinded to allocation, but the outcome assessor of the wound area was. Patients were allocated by block randomization.

Participants in the intervention group completed eight weeks with sessions of passive leg movement with both legs. During the eight-week trial period, all participants, in both the control and intervention groups, were examined four times (baseline and weeks 3, 5, and 8). After the trial period, there was one last follow-up visit at week 16. The primary outcome measure was wound area and wound area reduction. Secondary outcome measures were full epithelialization of the wound and Meggitt-Wagner score. Patients, who developed a deep infection that needed intravenous antibiotics or amputation between baseline and week 8, were excluded.

The study was conducted in the Capital Region of Denmark at Herlev Hospital, a highly specialized in-hospital wound care unit, and registered at ClinicalTrials.gov (ID: NCT02785198). All primary and secondary outcomes were defined a priori. The study was terminated before time due to insufficient recruitment of participants.

The study was approved by the Danish Medical Research Ethics Committee (approval number: H-15008102) and the Danish Data Protection Agency (journal number: 03953 and ID number: HGH-2015-023).

Participants

Patients with non-healing diabetic foot ulcers were recruited from three different highly specialized in-hospital wound care units in the greater Copenhagen area in Denmark: the Department of Orthopedic Surgery, Herlev Hospital, Steno Diabetes Center Copenhagen, and the Department of Orthopedic Surgery, Hvidovre University Hospital. A total of 194 patients were screened for eligibility, and 21 patients were enrolled between May 2016 and February 2018 by on-site investigators using the eligibility criteria listed below. All participants received oral and written information regarding the study and signed a written consent form before participating.

Diabetic patients with non-healing foot ulcers were eligible if they had the following: (1) diabetes mellitus according to the World Health Organization criteria ((a) fasting venous plasma glucose ≥7 mmol/l, (b) two-hour plasma glucose ≥11.1 mmol/l after the ingestion of 75 g oral glucose, or (c) HbA1c ≥6.5% (48 mmol/mol)) [23,24], (2) foot ulcer size >0.4 cm diameter/12.6 mm^2^, (3) clinically non-healed foot ulcers >6 weeks, and (4) age >18 years.

Patients were excluded if they had the following: (1) major infection such as acute cellulitis, osteomyelitis, or gangrene anywhere in the affected extremity, (2) malignant disease (cancer requiring treatment), (3) major traumatic tissue damage (fractures, décollement), (4) major lower extremity amputation, and (5) dementia diagnosis or other cognitive impairment.

Experimental protocol

At the visits to the wound care unit at baseline and weeks 3, 5, 8, and 16, all participants received standardized wound treatment. This included off-loading of the wound, debridement, dressings, compression, and local or oral antibiotics if necessary. Furthermore, the wound was described according to the Meggitt-Wagner classification, a digital photograph was taken, and the participants in the intervention group conducted one session of passive movement.

At baseline, the participants underwent a distal toe and ankle pressure measurement, a distal perfusion pressure measurement at foot level, and two functional tests: the sit-to-stand test and the timed up-and-go test.

The wound area was calculated using digital planimetry (Fiji ImageJ 1.49). The wound edges were manually traced in the program, and the wound area was calculated in mm^2^. The Meggitt-Wagner classification and wound area calculations were conducted by a blinded secondary investigator.

Training protocol and passive movement model design

**Figure 1:**
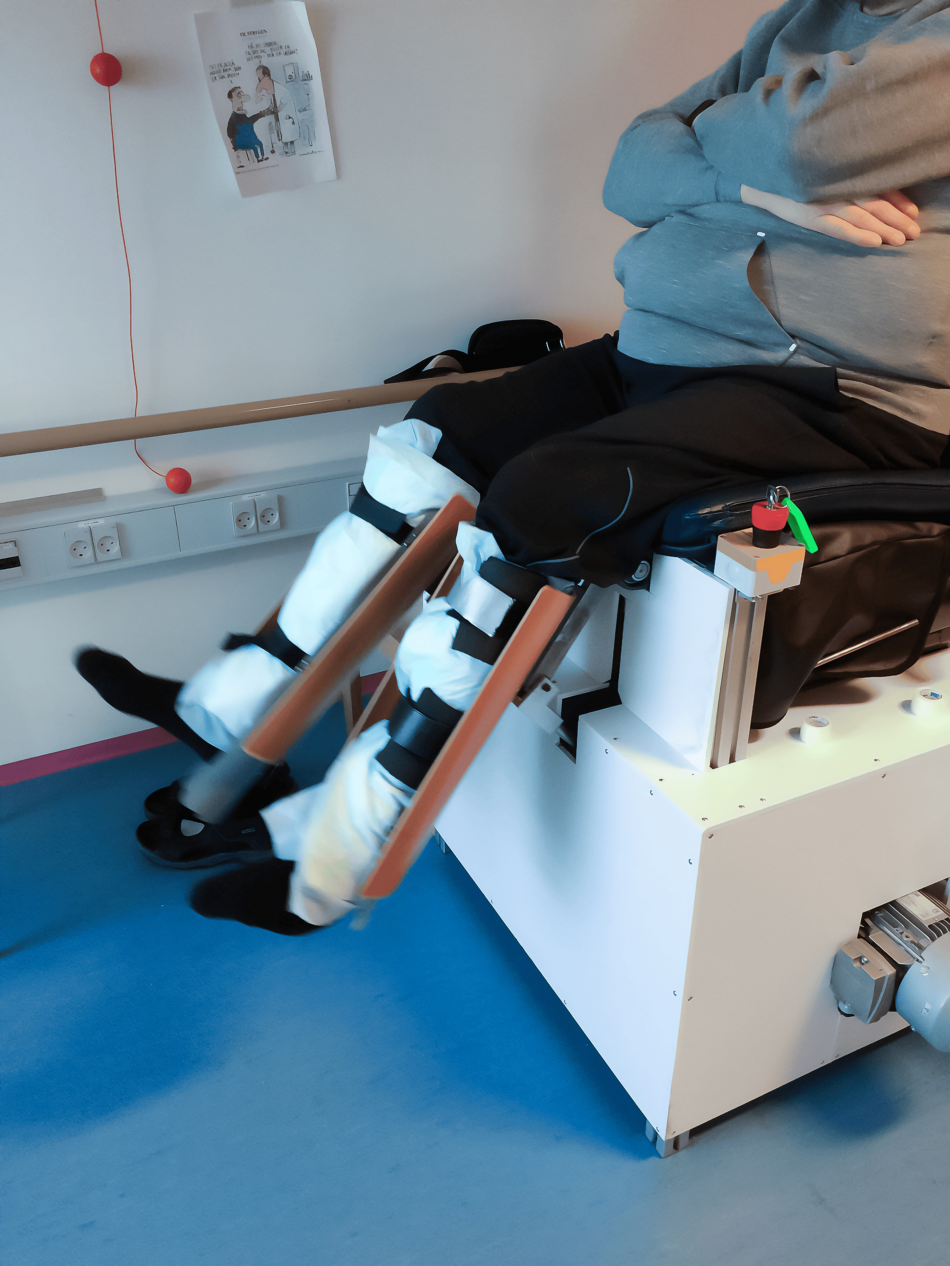
The passive movement model

The intervention consisted of passive knee extensions of both legs, at a range of motion of 60 degrees, at a frequency of 60 extensions and flexions per minute. The movement was conducted in a specially designed machine: the passive leg movement machine (Figure 1). The training sessions were supervised and conducted three times per week for eight weeks, and each session lasted 60 minutes.

The passive leg movement machine was made in collaboration with the Technical University of Denmark (DTU) and inspired by the one-leg kicking model developed at the Department of Nutrition, Exercise, and Sports, University of Copenhagen, Copenhagen, Denmark. The passive leg movement machine was designed to be motorized and to move both legs (Figure 1).

Data analysis

The null hypothesis was that there was no difference in wound healing between the intervention and the control group after eight weeks. With a power of 0.8, a standard deviation of 0.4, and a hypothesized difference of 0.35 between the two groups, the sample size was calculated to include 44 participants in total. The level of significance was set to 0.05. The research group chose 0.35 (35%) wound area reduction as the clinically significant difference between the two groups, because the difference between groups should be of a certain size for it to be worth the patients' time, as they spend three hours per week in the machine for eight weeks.

Statistical analyses were performed using SAS Enterprise Guide 7.1 (SAS Institute Inc., Cary, North Carolina, United States). All parametric data are expressed as means including standard error of means (SEM) unless otherwise stated. Baseline and endpoint comparisons were made using an independent t-test. Binomial data was compared using Fisher's exact test. Correlations between different variables (infection parameters, wound duration, and timed up-and-go test/sit-to-stand test) on wound healing were calculated using Spearman's correlation coefficient. The strength of the correlations was classified into very weak (0.00-0.19), weak (0.20-0.39), moderate (0.40-0.59), strong (0.60-0.79), or very strong (0.80-1.00) [25]. Statistical significance was set at p<0.05.

Randomization was calculated by a statistician using R. Participant numbers were divided into 10 different block sizes, before randomizing each unique participant number to either passive training or control group. The results were put into anonymous envelopes with the participant number on the outside of the envelope, making it impossible for the doctor, who included the patient, to break the randomization.

## Results

**Figure 2:**
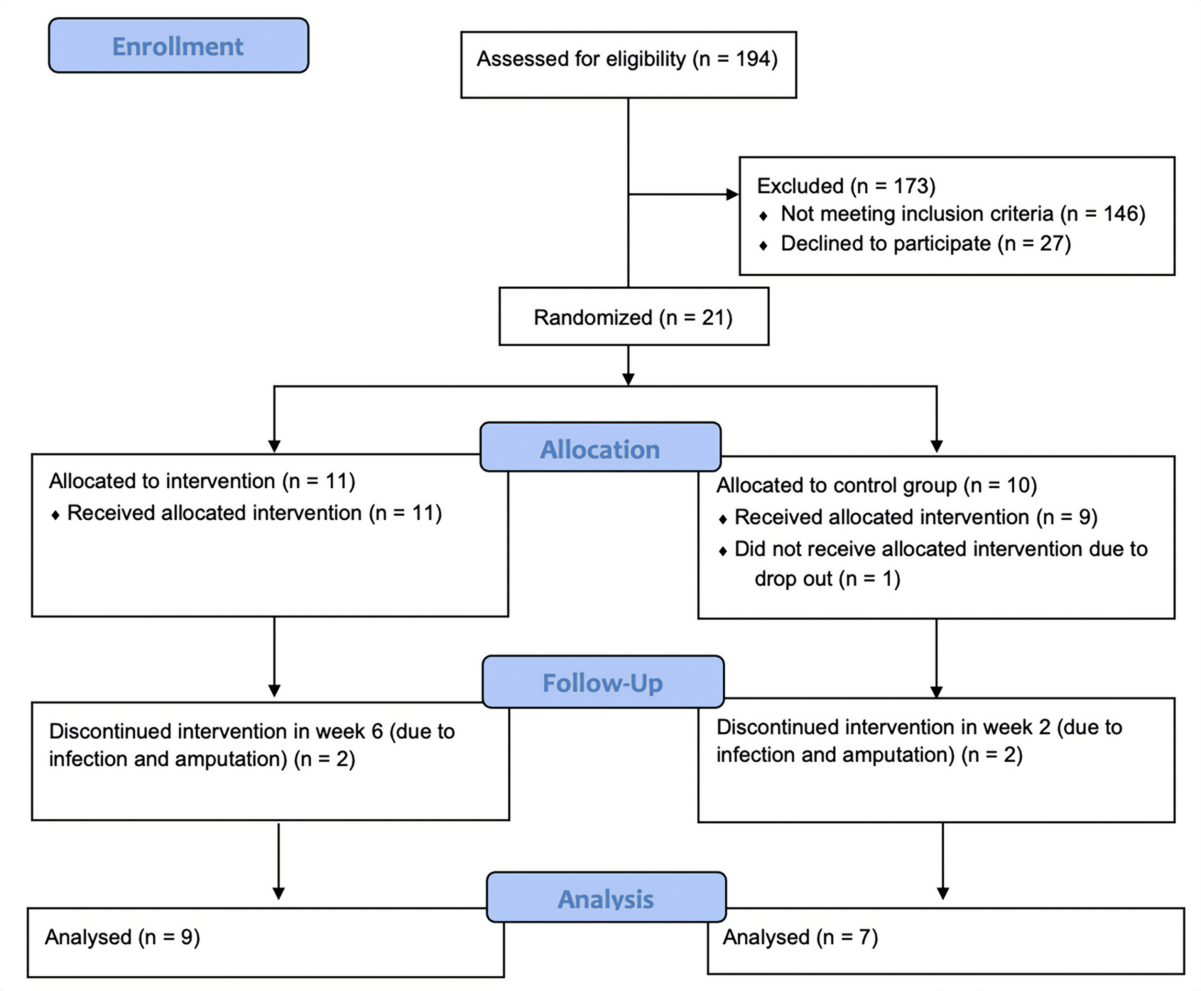
Flowchart for the enrollment of patients after CONSORT guidelines CONSORT: Consolidated Standards of Reporting Trials

A total of 21 diabetic patients with non-healing ulcers were included in the study: 11 participants in the intervention group and 10 participants in the control group. Two and three participants from the intervention and control groups, respectively, were excluded during the eight-week intervention period due to adverse events (Figure 2). The baseline characteristics of the two groups are listed in Table 1.

**Table 1:** Baseline characteristics of the participants Expressed in numbers (no.) or mean±SEM HbA1c: glycated hemoglobin; SEM: standard error of the mean

	Intervention group	Control group
No. of participants	11	10
Age (years)	58±1.7	64±4.8
Gender (male/female)	10/1	7/3
Body mass index (kg/m^2^)	29±0.9	28±2
Diabetes (no. type 1/no. type 2)	1/10	1/9
Diabetes duration (years)	17±3.2	25±3.7
HbA1c (mmol/mol)	65±4.2	60±6.6
Wound duration (weeks)	29±9.4	37±10.5
Meggitt-Wagner score (grade 1/2/3)	9/1/1	8/1/1
Wound area (mm²)	274±79.6	148±63.2
No. of patients with neuropathy	9	10
Ankle-brachial index	0.96±0.1	1.06±0.1
C-reactive protein (mg/L)	7±1.8	19±2.1
Leucocytes (×10^9^/L)	7.6±0.5	8.1±0.4
Glomerular filtration rate (ml/min/1.73 m^2^)	69±7.2	71±10.7
No. of patients with alcohol abuse (current and present)	7	1
No. of patients smoking or with a history of smoking	8	4
Sit-to-stand test (repetitions)	9.5±0.9	9±1.5
Timed up-and-go test (seconds)	12.8±1.6	13.2±2.8

Adverse events

No adverse events were recorded in relation to the passive movement training. Four adverse events were recorded in total. Two patients in the intervention group were excluded in week 6 due to deep infection in relation to their foot ulcer. In one of these two patients, the infection resulted in the amputation of the first toe, and the other patient was treated with debridement and intravenous antibiotics. Two patients from the control group were excluded in week 2, both due to deep wound infection, resulting in an above-knee amputation in one patient and an amputation of the first toe in the other.

Three out of seven patients in the control group underwent amputation between weeks 8 and 16, whereas only one out of nine participants in the intervention group underwent amputation during the follow-up period.

All nine participants in the intervention group completed the planned 24 passive exercise sessions.

Wound healing

**Figure 3:**
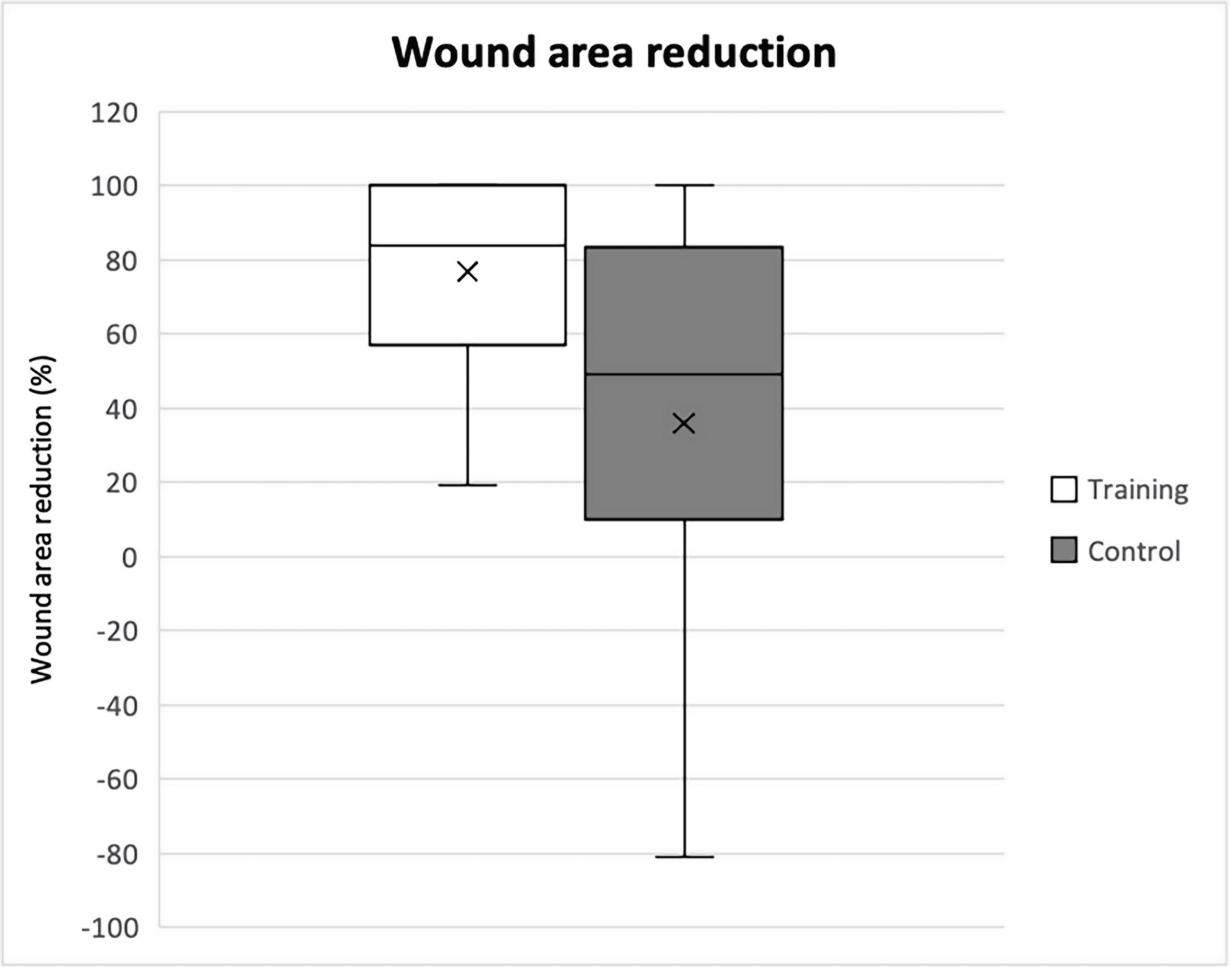
Wound area reduction in percent between baseline and week 8 Expressed in medians and interquartile ranges

Wound sizes for the intervention group were 274 mm^2^, 58 mm^2^, and 7 mm^2^ at baseline, week 8, and week 16, compared to 148 mm^2^ (p=0.31), 136 mm^2^ (p=0.51), and 23 mm^2^ (p=0.55) in the control group (Table 2). The mean wound area percentual reduction between baseline and week 8 was greater in the intervention group (76% in the intervention group vs. 36% in the control group, difference 40%, p=0.062), but this was not statistically significant (Figure 3). Three participants in the intervention group (33.3%) showed total wound epithelialization at week 8 versus one participant in the control group (14.3%). At the follow-up visit at 16 weeks, five out of nine participants (55.6%) showed total wound closure in the intervention group, compared to three out of seven (42.9%) in the control group (Table 3).

**Table 2:** Wound area at baseline, week 8, and week 16

	Intervention group, mm^2^	Control group, mm^2^	P-value
Baseline	274	148	0.31
Week 8	58	136	0.51
Week 16	7	23	0.55

**Table 3:** Number of patients with full epithelialization/wound closure at eight and 16 weeks by groups in numbers (no.) and percentages

	Intervention group		Control group		P-value
	No.	Percentage	No.	Percentage	
8 weeks	3/9	33.3%	1/7	14.3%	0.59
16 weeks	5/9	55.6%	3/7	42.9%	1.00

There was no change in the Meggitt-Wagner score between baseline and week 8 in both groups.

There was a moderate, positive correlation between wound duration at baseline and wound area reduction between baseline and week 8 (r_s_=0.52, n=16, p=0.037). There was no correlation between wound area reduction and infection parameters or functional test results.

## Discussion

Our study evaluated the effect of eight weeks of passive movement on wound healing. There was a non-significant improvement in wound healing with the passive movement intervention, the passive movement exercise was well tolerated by the patients, and the adherence to training was excellent. Wound healing was assessed by change in the wound area, with a 40% greater wound area reduction in the intervention group compared to the control group. However, this might be due to chance and a type 1 error. A consensus report suggested that wound area measurements should be recorded as total closure (epithelialization) of the wound [26]. On this secondary outcome, we also saw a higher percentage of complete epithelialization in the intervention group, though this could also be due to chance or confounding.

To our knowledge, no previous studies have investigated the effect of passive movement training on wound healing in diabetic patients. However, one pilot study by Flahr [27] observed improved wound healing in diabetic patients after a non-weight-bearing foot exercise program, though the results were not statistically significant. A randomized controlled trial by Eraydin and Avşar [15] also showed improved wound healing after a non-weight-bearing foot exercise program, but this study was not blinded, which introduces a risk of bias. This intervention type can be compared to passive movement by its low training intensity.

Two feasibility trials [28,29], concerning non-weight-bearing exercise programs with stationary biking and strength training for patients with diabetic foot ulcers, indicated that a supervised non-weight-bearing training modality could be used for patients with active foot ulcers. Similar to our study, the training was tolerated well, and the adherence to training was adequate. However, none of the two studies included enough patients to show the true benefits on wound healing. Additionally, a randomized controlled trial [14] showed significantly improved wound healing in patients with type 2 diabetes with stationary biking, but this study was also not blinded.

Previous studies have shown that active exercise programs significantly improve wound healing of non-healing ulcers [30,31], and a recent systematic review by Tran and Haley concluded that, given the potential benefits of exercise on patient health and well-being, non-weight-bearing exercise should be encouraged as part of the management plan for the treatment of diabetic foot ulcers [32]. The advantage of passive movement over active exercise for this patient group is the lack of mechanical impact on the wound area. Thus, the passive movement may serve to bring the patient to an improved stage of healed ulcers, allowing for the initiation of an active exercise program.

Study limitations

The present study was underpowered, and therefore, further studies examining the promising effect of passive movement in this patient group are needed. Furthermore, despite randomization, some factors were unevenly distributed between the intervention and control groups, which may have caused confounding by these factors. Specifically, there were great differences between the two groups regarding wound duration, wound area, smoking, and alcohol intake. Thus, the interpretation of the results should take this into account.

## Conclusions

The two-leg passive movement intervention showed a non-statistically significant improvement in wound healing in patients with diabetic foot ulcers. Furthermore, the intervention was well tolerated by the patients and did not lead to any adverse events. Unfortunately, the study was terminated before full patient inclusion, and factors such as wound duration, wound area, smoking, and alcohol intake were unevenly distributed between groups, so the results should be interpreted with its limitations of being underpowered and potentially confounded. Yet, insights from this study can be used for larger research interventions in the future, examining the use of the passive movement modality for the treatment of diabetic foot ulcers.

## References

[1] (2023). Diabetes. https://www.who.int/health-topics/diabetes.

[2] Raja JM, Maturana MA, Kayali S, Khouzam A, Efeovbokhan N (2023). Diabetic foot ulcer: a comprehensive review of pathophysiology and management modalities. World J Clin Cases.

[3] Vas P, Rayman G, Dhatariya K (2020). Effectiveness of interventions to enhance healing of chronic foot ulcers in diabetes: a systematic review. Diabetes Metab Res Rev.

[4] McDermott K, Fang M, Boulton AJ, Selvin E, Hicks CW (2023). Etiology, epidemiology, and disparities in the burden of diabetic foot ulcers. Diabetes Care.

[5] Barnes JA, Eid MA, Creager MA, Goodney PP (2020). Epidemiology and risk of amputation in patients with diabetes mellitus and peripheral artery disease. Arterioscler Thromb Vasc Biol.

[6] Lin C, Liu J, Sun H (2020). Risk factors for lower extremity amputation in patients with diabetic foot ulcers: a meta-analysis. PLoS One.

[7] Polikandrioti M, Vasilopoulos G, Koutelekos I (2020). Quality of life in diabetic foot ulcer: associated factors and the impact of anxiety/depression and adherence to self-care. Int J Low Extrem Wounds.

[8] Dias Â, Ferreira G, Vilaça M, Pereira MG (2022). Quality of life in patients with diabetic foot ulcers: a cross-sectional study. Adv Skin Wound Care.

[9] Jodheea-Jutton A, Hindocha S, Bhaw-Luximon A (2022). Health economics of diabetic foot ulcer and recent trends to accelerate treatment. Foot (Edinb).

[10] Raghav A, Khan ZA, Labala RK, Ahmad J, Noor S, Mishra BK (2018). Financial burden of diabetic foot ulcers to world: a progressive topic to discuss always. Ther Adv Endocrinol Metab.

[11] Soyoye DO, Abiodun OO, Ikem RT, Kolawole BA, Akintomide AO (2021). Diabetes and peripheral artery disease: a review. World J Diabetes.

[12] Wang X, Yuan CX, Xu B, Yu Z (2022). Diabetic foot ulcers: classification, risk factors and management. World J Diabetes.

[13] Keylock T, Meserve L, Wolfe A (2018). Low-intensity exercise accelerates wound healing in diabetic mice. Wounds.

[14] Nwankwo MJ, Okoye GC, Victor EA, Obinna EA (2014). Effect of twelve weeks supervised aerobic exercise on ulcer healing and changes in selected biochemical profiles of diabetic foot ulcer subjects. Int J Diabetes Res.

[15] Eraydin Ş, Avşar G (2018). The effect of foot exercises on wound healing in type 2 diabetic patients with a foot ulcer: a randomized control study. J Wound Ostomy Continence Nurs.

[16] Wong E, Backholer K, Gearon E, Harding J, Freak-Poli R, Stevenson C, Peeters A (2013). Diabetes and risk of physical disability in adults: a systematic review and meta-analysis. Lancet Diabetes Endocrinol.

[17] Aagaard TV, Moeini S, Skou ST, Madsen UR, Brorson S (2022). Benefits and harms of exercise therapy for patients with diabetic foot ulcers: a systematic review. Int J Low Extrem Wounds.

[18] Høier B, Rufener N, Bojsen-Møller J, Bangsbo J, Hellsten Y (2010). The effect of passive movement training on angiogenic factors and capillary growth in human skeletal muscle. J Physiol.

[19] Hoier B, Walker M, Passos M (2013). Angiogenic response to passive movement and active exercise in individuals with peripheral arterial disease. J Appl Physiol (1985).

[20] Hellsten Y, Rufener N, Nielsen JJ, Høier B, Krustrup P, Bangsbo J (2008). Passive leg movement enhances interstitial VEGF protein, endothelial cell proliferation, and eNOS mRNA content in human skeletal muscle. Am J Physiol Regul Integr Comp Physiol.

[21] Martin A, Komada MR, Sane DC (2003). Abnormal angiogenesis in diabetes mellitus. Med Res Rev.

[22] Nyberg M, Gliemann L, Hellsten Y (2015). Vascular function in health, hypertension, and diabetes: effect of physical activity on skeletal muscle microcirculation. Scand J Med Sci Sports.

[23] (2006). Definition and diagnosis of diabetes mellitus and intermediate hyperglycaemia.

[24] (2011). Use of glycated haemoglobin (‎HbA1c)‎ in diagnosis of diabetes mellitus.

[25] (2023). 11. Correlation and regression. BMJ Lead. Gen. Med. J.

[26] Jeffcoate WJ, Bus SA, Game FL, Hinchliffe RJ, Price PE, Schaper NC (2016). Reporting standards of studies and papers on the prevention and management of foot ulcers in diabetes: required details and markers of good quality. Lancet Diabetes Endocrinol.

[27] Flahr D (2010). ﻿﻿The effect of nonweight-bearing exercise and protocol adherence on diabetic foot ulcer healing: a pilot study. Ostomy Wound Manage.

[28] Aagaard TV, Lindberg K, Brorson S, Madsen UR, Skou ST (2023). A 12-week supervised exercise therapy program for patients with diabetic foot ulcers: program development and preliminary feasibility. Int J Low Extrem Wounds.

[29] Lindberg K, Møller BS, Kirketerp-Møller K, Kristensen MT (2020). An exercise program for people with severe peripheral neuropathy and diabetic foot ulcers - a case series on feasibility and safety. Disabil Rehabil.

[30] Emery CF, Kiecolt-Glaser J, Glaser R, Malarkey WB, Devor S (2017). Aerobic exercise accelerates wound healing among stressed older women, but not stressed older men. Innov Aging.

[31] Goh J, Ladiges WC (2014). Exercise enhances wound healing and prevents cancer progression during aging by targeting macrophage polarity. Mech Ageing Dev.

[32] Tran MM, Haley MN (2021). Does exercise improve healing of diabetic foot ulcers? A systematic review. J Foot Ankle Res.

